# Let’s (not) Go Outside: Grindr, Hybrid Space, and Digital Queer Neighborhoods

**DOI:** 10.1007/978-3-030-66073-4_9

**Published:** 2020-11-30

**Authors:** Sam Miles

**Affiliations:** 17 Department of Architecture and Design, Alfred State University of New York, New York, USA; 18 Department of Urban and Regional Planning, University at Buffalo, Buffalo, NY USA; Public Health, Environments and Society, London School of Hygiene & Tropical Medicine, London, UK

**Keywords:** Technology, Geography, Hybridization, Space, Location-based media, Coronavirus

## Abstract

Developments in mobile digital technologies are disrupting conventional understandings of space and place for smartphone users. One way in which location-based media are refiguring previously taken-for-granted spatial traditions is via GPS-enabled online dating and hook-up apps. For sexual minorities, these apps can reconfigure any street, park, bar, or home into a queer space through a potential meeting between mutually attracted individuals, but what does this signify for already-existing queer spaces? This chapter examines how smartphone apps including Grindr, Tinder, and Blued synthesize online queer encounter with offline physical space to create a new hybrid terrain predicated on availability, connection, and encounter. It is also a terrain that can sidestep established gay neighborhoods entirely. I explore how this hybridization impacts on older, physically rooted gay neighborhoods and the role that these neighborhoods have traditionally played in brokering social and sexual connection for sexual minorities. Few would deny that location-based apps have come to play a valuable role in multiplying opportunities for sexual minorities. However, the stratospheric rise of these technologies also provokes questions about their impact on embodied encounter, queer community, and a sense of place. A decade on from Grindr’s release, this chapter evaluates the impact of location-based media on gay spaces and reflects on what the increasing hybridization of online and offline spaces for same-sex encounter might mean for queer lives of the future.

## Introduction

*Let’s go outside (let’s go outside),In the moonshineTake me to the places that I love best*

George Michael, *Outside* (1998)

In October 1998, mere months after his arrest for ‘engaging in a lewd sex act’ with another man in a Los Angeles park, singer George Michael released the hit single ‘Outside’, a musical celebration of sex in public. ‘I think I’m done with the sofa/I think I’m done with the hall/I think I’m done with the kitchen table, baby’, the singer suggests, before confessing: ‘You see I think about it all the time, 24/7’. The song concludes with a knowing wink to other cruisers: ‘Keep on funkin’, just keep on funkin’’. As a musical riposte to heteronormativity, the message was clear: queer life is best lived outside.

Two decades later, queer life is happening rather more inside, and when it comes to gay neighborhoods more widely, things are changing fast. Developments in mobile digital technologies over the past decade are refiguring previously taken-for-granted spatial traditions in today’s towns and cities in ways that incorporate online spaces more than ever before. One way in which this shift is occurring is via online dating, sex, and hook-up apps. The US digital dating app market alone is worth nearly $1 billion (Clement 2020), with disproportionately high LGBTQ subscription: 65% of same-sex couples now meet their partner online rather than in person, against 39% of heterosexual couples (Rosenfeld et al. 2019). Location-based media—that is, products that utilize the GPS location-sensing technologies offered by today’s smartphones^1^—now comprise the dominant platform for partner seeking across the global North. Male-male offerings including Grindr, Hornet, Scruff, and Blued, and female-female platforms including HER and Lex, as well as more mainstream apps increasingly utilized for same-sex searching such as Tinder and Badoo, have proven popular for both socialization and sexual encounter (Ahlm 2017; Ferris and Duguay 2020; Mearns 2020; Miles 2018).

Same-sex partner-seeking platforms, of which Grindr is the (in)famous market leader with users in 234 countries worldwide (Grindr 2020), have enjoyed particularly high adoption by gay, bisexual, and other men who seek sex with men. Membership of these platforms has become the norm not just throughout wealthy cities in North America and Europe, but also surprisingly widely around the world, including within sociopolitical cultures popularly perceived to be sexually conservative (Dasgupta 2017; Miao and Chan 2020) and economically marginalized settings in the global South (Birnholtz et al. 2020; Bryan 2019). This rapid and widespread shift to online, smartphone-enabled partner-seeking generates significant implications for offline gay neighborhoods, compounded by the economic impact of the 2020–21 coronavirus pandemic on what are, in many cities, already struggling queer commercial and community venues. For the LGBTQ app user, Grindr or Scruff or HER can reconfigure any street, park, bar, or home into a queer space by brokering a meeting between mutually attracted individuals. What, then, might this signify for already-existing queer spaces? Or to put it another way: what does a gay neighborhood look like today when *any* bar can constitute a gay bar for those meeting through location-based platforms? And how might this technologically hybridized route to encounter shape gay neighborhoods of the future?

For the purpose of this chapter I define hybridization as the layering, synthesizing, or collapsing of digital and physical realities. The result is a hybrid reality, landscape, or place, in this scenario for navigation by the dating app user seeking to make contact with a new partner(s). This chapter calls upon ideas of technological hybridization to explore how dating and hook-up apps synthesize online queer spaces of connection with offline, in-person meetings between interested parties—in what follows, primarily men, given their disproportionately high subscription to location-based apps.^2^ I also explore possible impacts of this hybridized encounter on older, physically rooted gay neighborhoods, and I reflect on future scenarios for online and offline neighborhoods in a post-coronavirus pandemic urban landscape. Few would deny that GPS-enabled apps have come to play a significant role in multiplying opportunities for sexual minorities; however, their unprecedented rise in popularity equally provokes questions about their impact on embodied encounter, community and a spatially oriented sense of place. When it comes to research, debates percolating around online self-presentation in queer technology use are now well-established (see for example Anderson et al. 2018; Bonner-Thompson 2017; Callander et al. 2015; Conner 2019; Miles 2019), but sustained examination of the lived, applied realities for these technology users in a digitally ‘enhanced’ but demonstrably physical context are still relatively limited. This chapter offers just one approach to how we might think about the changing relations between gay neighborhoods, communities, and mobile technologies.

Before going any further, there is a point here that needs to be emphasized regarding technological change. The location-based media landscape is continually evolving, and industry behemoths such as Grindr and Tinder that dominate today may in the near future be replaced by competitors, which will themselves be replaced over time by yet newer upstart platforms. Technological research is characterized by seemingly ever-changing developments, but the wider analyses offered in this chapter of how contemporary digital platforms impact on gay neighborhoods will hold true for technologies of the future, much as the patterns I explore here themselves echo interactions with desktop programs of the 1990s, from Yahoo listservs to Gaydar and PlanetRomeo, seen at the time as pioneering technological offerings (Miles 2018; Mowlabocus 2010). Understanding today’s platforms and how they function for users usefully informs exploration of related (and indeed seemingly unrelated) technologies of the present and future, even as the products themselves change. Indeed, the growing ‘digital turn’ in urban geography (Ash et al. 2018; Barns et al. 2017; Datta 2018; Engin et al. 2020; Kitchin 2014) strongly suggests that technological processes will become ever more dominant in our epistemological and empirical studies of urban life.

## Situating Sexualities, Cities, and Technologies

Understandings of space as a conventional cartography have been superseded in the last quarter-century by more humanistic and relational interpretations of space as flexible, multiple, and continually produced (Harvey 1989; Lefebvre 2004; Thrift 2006). Spaces are also sites of political, cultural and social negotiations and re-negotiations between groups and individuals. The development of these more contested social constructions of space have allowed critics to explore the exclusionary spaces and segregated spaces that have so often characterized queer urban life, from the social—and therefore spatial—primacy granted to heterosexual family life (Edelman 2004) to the red-light zones to which queer life has often been relegated. Excluded or undesirable spaces have played host to countless gay neighborhoods, liminal zones, or informal settlements wherein sexual minorities have built alliances and communities with each other as well as with other marginalized groups (Berlant and Warner 1998; Brown 2009; Hartal 2017; Irazábal and Huerta 2016; Orne 2017; Ross and Sullivan 2012). The diversifying populations of these gay neighborhoods meant that they became ‘centres of community that welcomed “the other”’ (Bitterman 2020: 100), whether defined as such by socioeconomic status, ethnicity, class, health, or intersections of these identities. Thus, even as contemporary understandings of the ‘gayborhood’ increasingly pivot on capitalist endeavor, exclusionary wealth, and homonormativity, there exists a queerer history of these same spaces as representative of the physical manifestations of normative hegemonic forces that work to decenter minorities, and by association their practices, in public spaces. Or to put it a different way: even the most commodified present-day gayborhoods have grown from more radical roots.

**Fig. 9.1:**
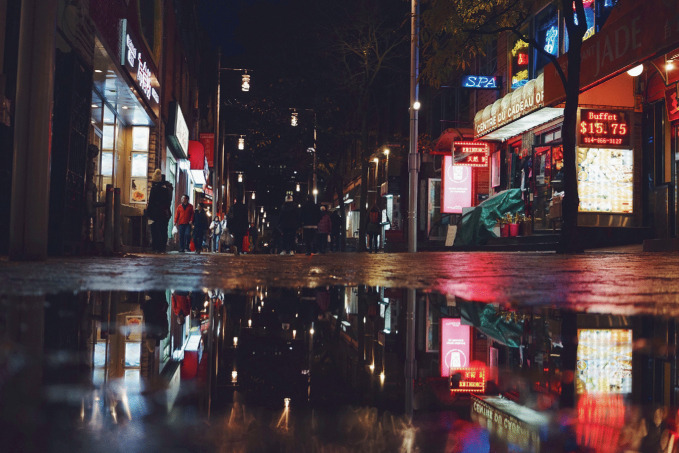
Urban streetscape at night (*Source* Guillame Jailleton for Unsplash (2017). Used with permission)

Certainly, while cities have constituted—and continue to constitute—spaces of sexual possibility, they are also sites upon which ‘ sexuality is most intensely scrutinised’ (Hubbard 2011: xiv). Given a history of surveillance, criminalization and homophobia and transphobia, sexual minorities have long had to negotiate and navigate both private and public spaces in complex and often subversive or dissident ways. As chapters elsewhere in this volume demonstrate (Eeckhout et al. 2021; Ghaziani 2021; Stone 2021), gay neighborhoods have developed over time as the spatial, generally urban manifestation of networks of sociability and solidarity between non-heterosexuals (Aldrich 2004; Gieseking 2020). Few would disagree with the idea that the city holds a particular cachet as a sexually stimulating environment (Bech 1997); within this environment, an historical synchronicity between urban terrain, sex, and sexuality, from cruising to commercial venues, still dominates today (Fig. 9.1).

To this distinctive history of place-making, technological hybridization has come to play a growing role in everyday queer life. Technology has become deeply incorporated into our lived environment. With an estimated 4.4 billion internet users worldwide (Kemp 2019), the internet has for many become ‘part of everyday life and sexuality’ (Johansson 2007: 118), and for many LGBTQ populations, including a sizeable proportion of gay, bisexual and other MSM, the integration of mobile and continuously connected internet into daily life has come to dominate sexuality and sexual practices. In the recent past, virtual worlds were considered distinct from ‘real’ spaces, but as technology has progressed in sophistication and portability, hybridization has developed as a more sustained relationship between the two entities. Technological hybridization challenges the assumption that digital space is predicated on transcending borders, boundaries, and geography to an ‘ Othered’ cyberspace. Instead, it offers an overlaying of physical environments with virtual connectivity and virtual and/or hybrid environments. The relationship between virtual and material worlds has become so intertwined as to now rarely be conceptualized as separate in any meaningful sense (see Barns et al. 2017; Kitchin and Dodge 2011; Farman 2012; Miles 2017). As Robyn Longhurst (2013: 667) argues: ‘people conduct their personal, familial, and emotional lives in a myriad of ways in a variety of different spaces. Bodies and spaces—cyber and ‘real’—are entangled’. These circulations generate pertinent questions about the way that we practice online life and what that looks like embedded in physical experience. Location-based media apps such as Grindr and Tinder offer a useful case study to witness some of these circulations in practice.

## Location-Based Dating Apps and Their Hybrid Queer Spaces

Central to the growth of digital-physical hybridization is the use of mobile phones, which are now the dominant platform for online connectivity worldwide (Clement 2019; O’Dea 2020). Contrary to anxieties raised by scholars including Zygmunt Bauman (2003) and Sherry Turkle (2011) regarding the negative implications of mobile virtual (un)reality on face-to-face communication, the reality borne out by technology users’ experiences seems to paint a significantly more relaxed picture. App users tend not to ‘escape’ or stop attending to their physical proximate environment due to their online connection(s); on the contrary, these location-based media figuratively overlay a user’s embodied reality with virtual connections with other people and places (see for example De Falco 2019; Gordon and de Souza e Silva 2011; Miles 2017; Race 2015).^3^ If we recognize the ability of the internet as a *broker* for embodied connections, the threat of unintentionally disconnecting from local territory is neutalized. Space again finds potential as something that can be practiced, imagined, and differently figured for each of its inhabitants, and this equally impacts on a physical sense of place. Whether this re-mediation of space and place via technology holds when it comes to a gay or queer neighborhood is less easily assumed, not least because ideas of what a gay neighborhood *is*, and its conceptual parameters, may actually be differently defined by different location-based media users, with conflicting attitudes and ambivalences (Miles 2017). Meanwhile, parallel debates permeate popular contemporary discourse: queer dating and hook-up apps are variously blamed for destroying gay neighborhoods and celebrated for reinvigorating them; dismissed as impediments to queer community by some and hypothesized by others as virtual sites for new and liberatory communities. Talking with users soon reveals that there are as many diverse attitudes to these apps as there are products themselves.

**Fig. 9.2:**
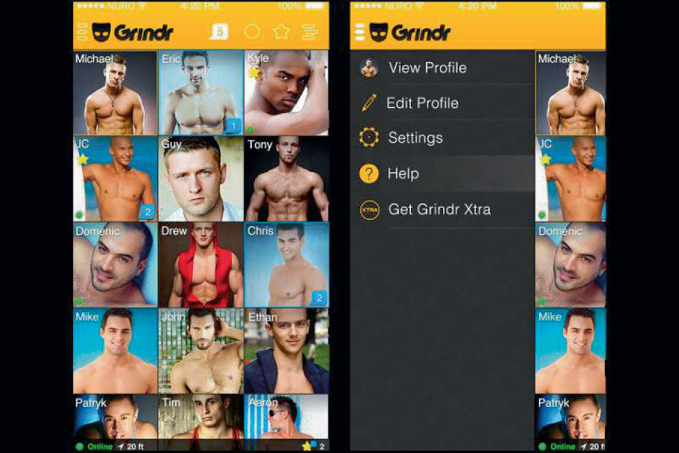
Grindr application (*Source* Grindr (2020). Used with permission)

**Fig. 9.3:**
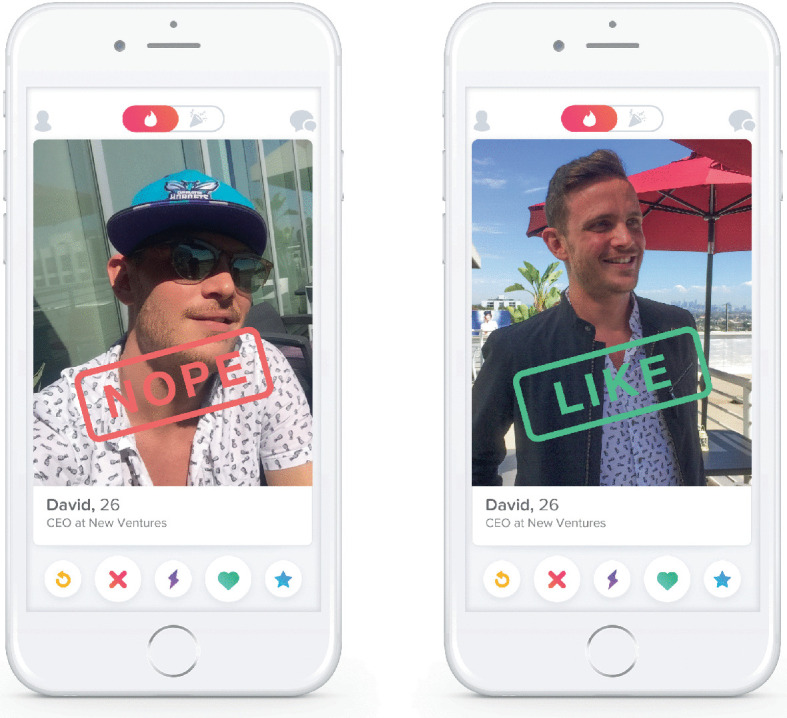
Tinder application (*Source* Tinder (2020). Used with permission)

The major attraction of apps such as Grindr, Hornet, and Blued that dominate online socialization for male-male encounter is their GPS mapping function. This feature pinpoints a user’s physical coordinates in order to filter potential matches by proximity, with the aim of expediting localized physical encounters developed from online introductions. By displaying a visual grid ( Grindr) or shuffled card deck ( Tinder) of potential matches for sex, relationships, and dating ordered by distance (see Figs. 9.2 and 9.3), these platforms streamline the process of meeting and allow the user to filter extensively for desired characteristics in any potential match: including, controversially, ethnicity and HIV status^4^ (Conner 2019; Lim et al. 2020; Shield 2018). In fact, visiting a town center, new neighborhood, or high street for the first time and loading the Grindr app makes for a curiously postmodern pastiche of cruising, parsing as it does the likeminded from the uninterested (Miles 2018). But these apps also mark a departure from the spontaneity of traditional ‘analog’ cruising. Dating apps allow the user to filter potential matches by age, body type (‘bear’,^5^ ‘ jock’, ‘geek’, ‘mature’), and distance before even an online introduction, let alone a physical encounter. These are algorithmically gifted digital matchmakers, and their filtering abilities are staggering.

 Queer men have long used subcultural codes, from fashion items like a single earring or colored handkerchiefs, to language and slang such as Polari, to assist in identifying each other in public. Now this peer identification is expedited through digital algorithms in users’ pockets and executed in real-time. Kane Race (2015: 271) captures the distinctive qualities of the spaces created by this location-based technology when he argues that apps are ‘participating in the construction of a specific sphere of sociability and amiable acquaintance among men in urban centers that prioritizes sex as a principle mechanism for connection and sociability’. Indeed, it is not unreasonable to suggest that these apps influence how users conceive of urban spaces and how they navigate for social or sexual opportunities in ways that echo traditional histories of flâneurie or homoerotic cruising in the metropolis (Turner 2003; Delany 1999), whether in terms of an initial shared gaze of interest or traversing the district or neighborhood in search of brief *physical* sexual contact. By opening up supposedly ‘ straight’ sites for queer encounter, these apps thwart the heteronormative status quo that often undergirds public urban space. Even the most intimidating sports bar can play host to a same-sex encounter, if the 4G reception allows. App users can thus use the technology in their hands to queer dominant norms in ways that can feel novel and refreshing when outside of established gay neighborhoods.

However, while this queer overlay of otherwise heterosexual space is in many ways welcome for its capacity for opening up new spaces and places for non-heterosexual encounter, it inevitably lessens the centrality of what were formerly go-to queer venues and neighborhoods. It is certainly worth thinking about ‘how gay men experience the division between dating apps and other online gay venues, and moreover, the division among user groups clustered around different dating apps’ (Wu and Ward 2017: 8). It is also pertinent to consider *which* sexual minorities are most able to capitalize on the potential of digital platforms for their partner seeking. As recent scholarship demonstrates, lesbian and queerer online partner-seeking networks suffer from some of the same marginalization as their physical counterpart spaces (Ferris and Duguay 2020; Duguay 2019; Murray and Ankerson 2016).

In addition to these inequalities, perhaps the yet more urgent need is to get a better grasp on how app users experience the division between dating apps and *offline* venues. More than their novel disruption of ostensibly heterosexual (and heterosexist) physical spaces with their virtual matchmaking abilities, these apps offer users the chance to find partners without needing to be physically rooted *in any kind of* *gay neighborhood* *at all*. David Harvey’s (1989) theory of time-space compression is usually applied to contemporary global flows, but if we conceptualize the city as a huge space to be processed and queer nightlife, for example, as a ‘portion’ of time, location-based apps compress the two variables so that from one spot on a night out the app user can survey thousands of meters in radius, and do so in mere seconds. This expert hybridization process not only matches interested partners and expedites physical meetings but also accelerates external factors in the privatization of queer space. Now any bar or restaurant can be a site for a first date; any home, hotel, or park can be a site for a sexual encounter. These spaces need not be gay bars or gay neighborhoods or gay saunas, because the obstacle of ascertaining mutual interest in a potential encounter has already been tackled and successfully overcome via the online scoping undertaken. Spaces with ‘gay inscriptions, both physical or symbolic, are not necessarily required’ (Visser 2013: 273). What *is* generated is a small gay neighborhood (indeed so small as to be in most cases dyadic, involving only (but not always) two people), with an entirely different ‘sense of place’. We turn now to consider the impact of these impromptu, digitally hybridized spaces on already-existing gay neighborhoods.

## The Ambiguous Impact of Location-Based Media on Existing Gayborhoods

 Location-based media are by no means the first digital intervention into physical same-sex encounter, not least because they echo partner-seeking apparatus popularly utilized in the 1990s via desktop listservs and static websites. Yet location-based media do seem to capture both the critical and cultural imagination when it comes to considering their impact on the health of gay neighborhoods. We have seen that dating and hook-up apps combine online queer encounters with offline physical space to synthesize a new hybrid terrain predicated on availability, connection, and erotic encounter. This is also a terrain that can sidestep established gay neighborhoods entirely. Consequently, the role that these neighborhoods have traditionally played in brokering social and sexual connection for sexual minorities is nullified. What then might this mean for gay neighborhoods and their value for same-sex encounter?

The first thing to consider is that cybersexual encounters are not always corporealized. Contact brokered online may stay online (Miles 2019), and there is no reason why these virtual connections cannot be richly fulfilling in and of themselves—emotionally, sexually, platonically, or politically. However, where cybersexual practices *are* converted to in-person meetings, whether pre-arranged or spontaneously, app users are increasingly meeting in private spaces, usually the home (Giraud 2016; Koch and Miles 2020). In the process, they sidestep certain risks generated by same-sex public meeting: anything from being harassed by passersby on a date to being bothered by police or security staff when cruising in public. Apps also negate the historical necessity of visiting queer entertainment venues to find and network with potential partners. This spatial shift at the hands of locative technology plays into what Michael Warner (1999: 153) had previously warned was a wider tendency to a ‘politics of privatization’, whereby mainstream social norms operate to restrict queer publics, either via assimilation to the norm or by pushing these publics out of sight altogether: in other words, play it ‘ straight’ and keep your kinky business at home. Unfortunately, the role of location-based media in compounding this kind of spatial privatization seems to suggest a capitulation to the heteronormative status quo rather than a generative queering of existing exclusionary spaces.

**Fig. 9.4:**
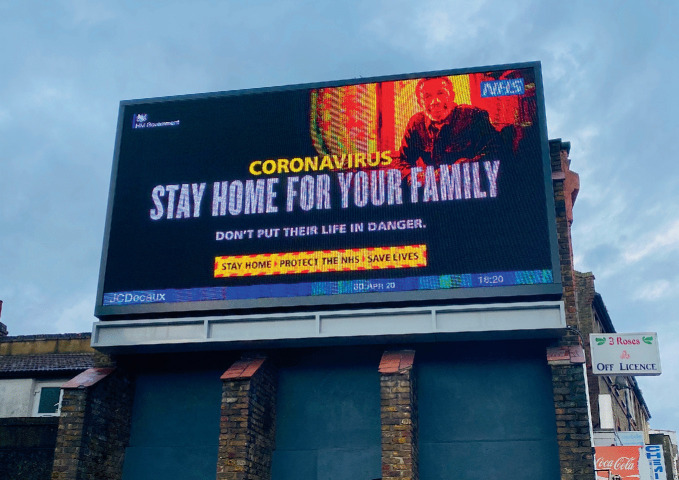
UK Government electric billboard campaign, London. Coronavirus: Stay home for your family (2020) (*Source* Image by author)

What this sidestepping of gay neighborhoods in turn means for the home is also worth considering. Private space provides a freedom that is often not tenable in public, and this is thrown into yet sharper relief in the context of a global health crisis. The staggering impact of the 2020-21 coronavirus pandemic has hugely restricted physical interpersonal interactions (Fig. 9.4), but where encounters have happened, they invariably occur in the private space of home and conversely less than ever in public commercial venues, which in many countries were shut down as a result of the virus’ spread—in some cases indefinitely. Even physical cruising is reconfigured, shifting from meetings in known physical areas to online introductions and meetings in the home. The results are mixed: cruising through location-based apps sidesteps potentially embarrassing false starts with non-queer subjects, but it also reduces serendipity (Miles 2018). By making physical meet-ups premeditated, with partner characteristics a known (and filtered) quantity, the chance of chance meeting on the street is drastically reduced. This process engineers out the unpredictability and *diversity* of potential street-level encounters in an embodied context. Indeed, while the domestication of formerly public encounters invites new forms of queer intimacy in the home, it extirpates the more positive elements of a gay neighborhood—a sense of community, a sense of collective safety, and for some, even a way of life.

Clearly, the ongoing diffusion of queer individuals from distinct ‘ gay villages’ to more scattered residential zones, and correlative decreases in LGBTQ commercial and community venues in cities of the global north (Gorman-Murray and Nash 2014; Kanai and Kenttamaa-Squires 2015; Mattson 2019; Podmore 2021; Whittemore and Smart 2016), may be attributable at least in part to location-based media (Collins and Drinkwater 2016; Gorman-Murray and Nash 2016; Roth 2016). The ability of digital technology to facilitate cybersexual encounter, hook-ups, and longer-term relationships online ab initio certainly seems to contribute to wider processes of change—whether displacement, movement, or deconcentration—of queer physical meeting-places in many cities of the global North. This is not to say that gay neighborhoods exist only to service partner-seeking, given that they perform a wide range of holistic and community roles. However, it *is* to say that partner-seeking is a not-insignificant part of the offering. Collins and Drinkwater (2016: 2) are unequivocal in their assessment that the ‘ubiquity of friend and partner search apps on smartphones have reduced the demand for, and thus rendered seemingly redundant, most smaller gay districts’. Nevertheless, they rightly caution against jumping to conclusions or making assumptions about the extent to which these apps are responsible for queer deconcentration. A more balanced (or ambiguous, depending on one’s ideological position) interpretation is that gay neighborhoods are not declining so much as shifting and changing, reflecting the organic (and often contested) status of these spaces more generally (Hess and Bitterman 2021; Doan and Atalay 2021; Ghaziani 2015; Hess 2019; Miles 2017; Renninger 2018). Perhaps spatial diffusions of queer culture away from gayborhoods ‘does not signal a destructive de-spatialization but rather a more dynamic series of ongoing  re-spatializations across a multitude of spaces’ (Bitterman 2020: 99). Further, while online sex and dating technologies may impact queer commercial venues, wider economic forces are more likely to have driven these urban changes (see Campkin and Marshall 2017; Lewis 2016; Mattson 2019, among others). Nevertheless, location-based technology has undoubtedly had an impact. As Hubbard et al. (2016: 568) argue:

While the significance of new technologies and the profusion of sexual content online can easily be overstated, there has clearly been something important happening here, with some of the traditional boundaries between private and public, intimate and shared, suburban and urban being inverted.

Because location-based media allow almost any space to constitute a queer space via their ‘plugged-in’ hybrid qualities, the primacy of existing urban venues such as gay bars for queer encounter is reduced. The question then becomes whether the attraction of the aforementioned commercial venues, along with community venues, queer residential clusters, and gayborhoods more widely is reduced. If technology users stop occupying these spaces (as restrictive or ‘ homonormative’^6^ as such spaces may be) in favor of online or private physical spaces, these queer spaces may diminish. For gay neighborhoods already undergoing deconcentration, the combination of neoliberal gentrification, acute economic shocks, location-based technology—and now the coronavirus pandemic—may foment a perfect storm for unmitigated decline. With it may well come the loss of more-than-concrete queer publics.

## Conclusion: Space for Co-Existence?

The exploration of contemporary digital media in this chapter is undertaken in the hope of providing more widely transferable ideas about spaces, communities, and technologies, and how these interact when it comes to gay neighborhoods in the near and more distant future. By better understanding the impact of location-based media on space and embodiment, we can make valuable inferences about a range of sexualities, practices, and urban environments. This chapter has explored how location-based technologies specifically impact queer male social and sexual encounters and queer physical spaces. It has argued that rather than displacing physical gay neighborhoods in a straightforward way, digital technologies hybridize online and offline encounters, imbuing any given physical locale with a potential queer connection while at the same time decentralizing the primacy of older, established gay neighborhoods. In this process, questions that arise about the centrality and durability of gay neighborhoods are valuable and deserve consideration. It may be true that for George Michael and others, it was the broadly defined ‘outside’—of the house, of the workplace, even of ‘the closet’—that offered the best freedom for gay expression (and indeed gay sex), but many decades of community building, queer commerce, and in some cities even urban planning have helped to develop physically defined gay neighborhoods with a wealth of attractions and minority protections. The loss of these hard-fought for, hard-won places and spaces seem inconceivable.

Yet looking forward, might there be space for partner-seeking apps *and* traditional gay neighborhoods? If so, success seems based on a conceptual shift from physical space as the *de rigueur* site for sexual encounter to something more of a holistic environment of safety and community. Certainly, case studies in a range of different cities suggest that gay neighborhoods are in flux, but that the outcomes need not be negatively assumed (Eeckhout et al. 2021; Ghaziani 2021; Coffin 2021; Podmore 2021). If today represents a ‘transitional stage toward a post-gay, post-binary-identity era’ (Hess 2019: 230), that is not to say that gayborhoods, however they are manifested, will not retain relevance as sites for community and safety for years to come—just that how they are manifested remains up for debate.

Relatedly, in the same way that paying greater attention to the formation of inner-city neighborhoods *beyond* the gay village which are increasingly associated with LGBTQ populations proves generative (Podmore 2021), appreciating and exploring the hybrid bricolage of online and offline queer life separate to its potential negative impact on bricks and mortar is also generative. For example, London’s Soho, with its long history of vice and permissiveness (Andersson 2009) historically functioned as the UK’s pre-eminent gay district, as a place in which gay identities are narrated and performed. Yet its booming tourism and rapidly rising property costs as a consequence of lucrative real-estate investment have diluted its queer presence in recent decades. Interviews with app users in Soho find that the cultural capital of a historically queer urban environment like Soho is conceptualized as a symbolic space of the past rather than a lived reality for many app users choosing to meet partners in local venues, ‘ straight’ venues, or in their own homes (Miles 2017). This shift is reflected stateside, by Jen Jack Gieseking’s study of lesbian and queer New York City (2020) and by Amy Stone’s (2020) case study of the heterosexualization of Baton Rouge’s Spanishtown, where consumption of historically gay culture by heterosexual parade participants generates an ambivalence about the space for LGBTQ citizens even as they participate in its festivities. In London’s Soho, app users’ emotional (and erotic) attachment to gay neighborhoods has not necessarily diminished so much as shifted into a space of queer social opportunities, and more ambivalently received international tourism (Miles 2017); yet in the faltering economic and touristic recovery wrought by the 2020–21 coronavirus pandemic, this shift may not be a bad thing. Meanwhile, Renninger (2018: 1737) finds that while app users seem cognizant of space and place in their app-facilitated encounters, ‘the use of these apps creates an attitude toward space that does not unblinkingly equate Grindr’s purpose with those of gay bars (and gayborhoods)’. Such a position suggests that apps such as Grindr overlap in purpose with these physical spaces, but not overwhelmingly so. There may therefore be space for both to exist in combination. Finally, Collins and Drinkwater (2016: 11) predict that ‘sexual and social community will, in effect, primarily reside in the online world but physically occupy mainstream social spaces whenever required’, but that occupation of mainstream social spaces may itself be imbued with a welcome queerness. These scenarios demonstrate hybrid potentialities for gay neighborhoods that may look and feel different to what has come before, yet have much to offer queer technology users and non-users alike.

There may be also room for a reconceptualization of what constitutes public space for app users. A conceptual shift seems to be occurring that moves gay and bisexual public spaces to domestic spaces of home, ostensibly at the hands of popular location-based apps that expedite and privatize the social or sexual encounter (Koch and Miles 2020). But perhaps we can rethink the encounters brokered by location-based media as not necessarily ‘private’ and not a wholesale rejection of ‘public’, but rather as a mixing of the two spheres. Public and private are not, after all, absolute categories (see Blunt and Sheringham 2019; Sheller and Urry 2003). In the same way that Ghaziani (2021: 87) challenges claims that ‘ gayborhoods as an urban form are outmoded or obsolete’, I would argue that by thinking more flexibly about how homes operate as spaces for queer encounter, hybridization can be conceptualized as a process that synthesizes not just digital and physical realms but also public and private spaces, recognizing in the process the increasingly blurred boundaries between these previously oppositional planes. Who is to say that the private home cannot constitute a gay neighborhood of sorts? It may be rather different from San Francisco’s Mission District or Madrid’s Chueca barrio, but that is not to say it cannot offer its own attractions.

Finally, we might think more flexibly about a post-gayborhood world. In a global context in which whole societies are still reeling from the impact of the coronavirus pandemic, the idea of gay neighborhoods as ‘post-places’ (Coffin 2021) feels positively funereal, but as Coffin argues (Coffin 2021: 373), ‘individuals and collectives may still be inspired by the memories, representations, and imaginaries previously provided by these erstwhile places.’ We are still absorbing the full impact of coronavirus on queer communities and on commercial venues which depend on close contact, in-person interaction and intimate socializing, but it may well prove to be the case that the ‘scene’ in many cities remains either temporarily or permanently muted. In such a scenario, digital technologies will offer a much-needed resource for queer encounter, for a strikingly wide range of users and communities. It seems likely that we will be met with a whole range of different spatial and conceptual configurations: the fresh air and ‘Outside’ of the George Michael pop song, and the ‘inside’ indoor life of a coronavirus lockdown; the ‘online’ space of a dating app and the ‘offline’ life of a gay bar—or all of these together, remixed and reformulated.

## Footnotes

1 Variously called locative media, Location-Based Social Apps (LBSAs) and People Nearby Applications (PNAs).

2 Though see for example Duguay (2019) for queer women’s’ experiences of ‘scarcity’ in geospatial partner-seeking.

3 This can hold in reverse, too: today’s smartphones contribute to an embodied experience within online space(s) from which they can influence a user’s affective, physical realm.

4 In June 2020, days into U.S. and then international Black Lives Matter protests, Grindr announced it would be removing its ethnicity filter in all versions of its app (Hern 2020).

5 Commonly referring to hairier, larger-bodied or more homomasculine men, but subjectively interpreted; as Yoel Roth (2014: 2122) argues, ‘defining these terms any more precisely than as sexual stereotypes is a task best left to the imaginations of individual users’.

6 ‘Homonormativity’ Duggan (2003) describes a depoliticized gay culture that valorizes domesticity and consumption, in the process sustaining heterosexual dominance in society.
